# Percutaneous balloon compression of trigeminal ganglion under conscious sedation local anesthesia for the treatment of primary trigeminal neuralgia—A prospective cohort study

**DOI:** 10.3389/fneur.2023.1144034

**Published:** 2023-05-17

**Authors:** Dou Zhi, Yuna Guo, Liangliang He, Liqiang Yang

**Affiliations:** Xuanwu Hospital, Capital Medical University, Beijing, China

**Keywords:** trigeminal neuralgia, balloon compression, local anesthesia, wide-awake, trigeminal ganglion

## Abstract

**Introduction:**

Trigeminal neuralgia is a debilitating condition that can significantly impair the quality of life of affected individuals. Percutaneous balloon compression (PBC) has been established as an effective treatment for this condition. However, the use of general anesthesia during the procedure poses challenges to achieving the desired degree of nerve damage without causing excessive numbness. In this study, we aimed to evaluate the feasibility and efficacy of performing PBC under conscious sedation of local anesthesia.

**Methods:**

We improved the surgical procedure for PBC by administering intraganglionic lidocaine 0.2% with fine needle aspiration to achieve conscious sedation. This allowed the operator to determine the degree of nerve damage in real time through the tactile test. We conducted a clinical observation of 87 patients who underwent PBC under conscious sedation of local anesthesia. We evaluated the intraoperative blood pressure and heart rate changes, postoperative facial pain relief, and occurrence of complications such as severe facial numbness, irreversible keratitis, vision loss, and masticatory muscle weakness.

**Results:**

All 87 patients achieved immediate relief of facial pain after undergoing PBC under conscious sedation of local anesthesia. The intraoperative blood pressure and heart rate changes were <20% of the baseline value. No patient experienced severe facial numbness or developed irreversible keratitis, vision loss, or masticatory muscle weakness.

**Discussion:**

Our findings suggest that PBC under wide-awake local anesthesia is a safe and effective treatment for trigeminal neuralgia. The use of conscious sedation of local anesthesia during the procedure allows the operator to achieve the desired degree of nerve damage without causing excessive numbness. This can lead to long-term pain relief and improved quality of life for patients with trigeminal neuralgia.

## Introduction

Trigeminal neuralgia (TN) is a medical condition characterized by a sudden, severe, stabbing pain that is usually confined to one side of the face and occurs in the distribution of one or more branches of the fifth cranial nerve. This definition is recognized by the International Association for the Study of Pain (IASP) (1). Balloon compression, a classic treatment method for TN, was first employed in clinical practice by Mullan and Lichtor in 1978. Related articles on the subject were subsequently published in 1983 (2).

As puncture and balloon compression during surgery for trigeminal neuralgia can lead to severe hemodynamic fluctuations, general anesthesia is commonly used to ensure patient safety. However, under general anesthesia, the surgeon is unable to receive feedback from the patient on the degree of hypoesthesia, leaving him to rely solely on experience to set balloon pressure and compression time. This often results in patients experiencing more numbness than intended (excessive nerve damage) or recurrence after surgery (insufficient nerve damage).

Huang et al. reported performing balloon compression surgery on patients under procedural sedation and analgesia using fentanyl and midazolam. This approach enables patients to remain moderately or lightly sedated during the procedure, resulting in a more appropriate degree of nerve damage (3). However, we believe that this method may not necessarily reduce the risk of trigemino-cardiac reflex (TCR) compared to general anesthesia.

Recent studies conducted by Tibano et al. (4) and Ararwal et al. (5) have shown that combining the trigeminal ganglion block with general anesthesia can significantly inhibit the TCR during balloon compression, thus reducing circulation fluctuations. Moreover, no significant complications associated with local anesthesia have been observed. This suggests that local anesthesia, which involves blocking sensory afferents of the trigeminal nerve, can reduce a patient's stress response more effectively than general anesthesia. Therefore, we believe that complete local anesthesia is sufficient to meet the analgesic needs of this surgery.

Based on the case series study, we conclude that percutaneous balloon compression of the trigeminal ganglion under conscious sedation local anesthesia is both effective and safe for the treatment of trigeminal neuralgia.

## Methods

### Patient population

This study was conducted with the approval of the Institution's Ethics Examining Committee of Human Research. Between August 2020 and September 2021, percutaneous balloon compression with local anesthesia was performed on 87 patients with trigeminal neuralgia. The age of the patients ranged from 39 to 86 years old, and the duration/diagnosis of TN ranged from 1 to 21 years.

The following were the inclusion criteria of the respondents: (1) those who met the diagnostic criteria for primary trigeminal neuralgia in accordance with the International Classification of Headache Disorders (6); (2) those who had a pain visual analog scale (VAS) score of ≥4; (3) those who had the duration of TN ≥ 6 months; and (4) those who had poor response to drug therapy or intolerable side effects.

Patients with the following conditions were excluded (7): (1) patients with severe systemic diseases who were intolerable to surgery and anesthesia, (2) coagulation dysfunction or local infection in the puncture area before surgery, (3) patients with mental illness, who were unable to express subjective feelings clearly, (4) patients who had undergone previous trigeminal nerve-related surgeries including microvascular decompression and nerve lesion, and (5) patients with reduced or abnormal superficial sensation in the affected facial area.

### Percutaneous balloon compression procedure

**Figure 1:**
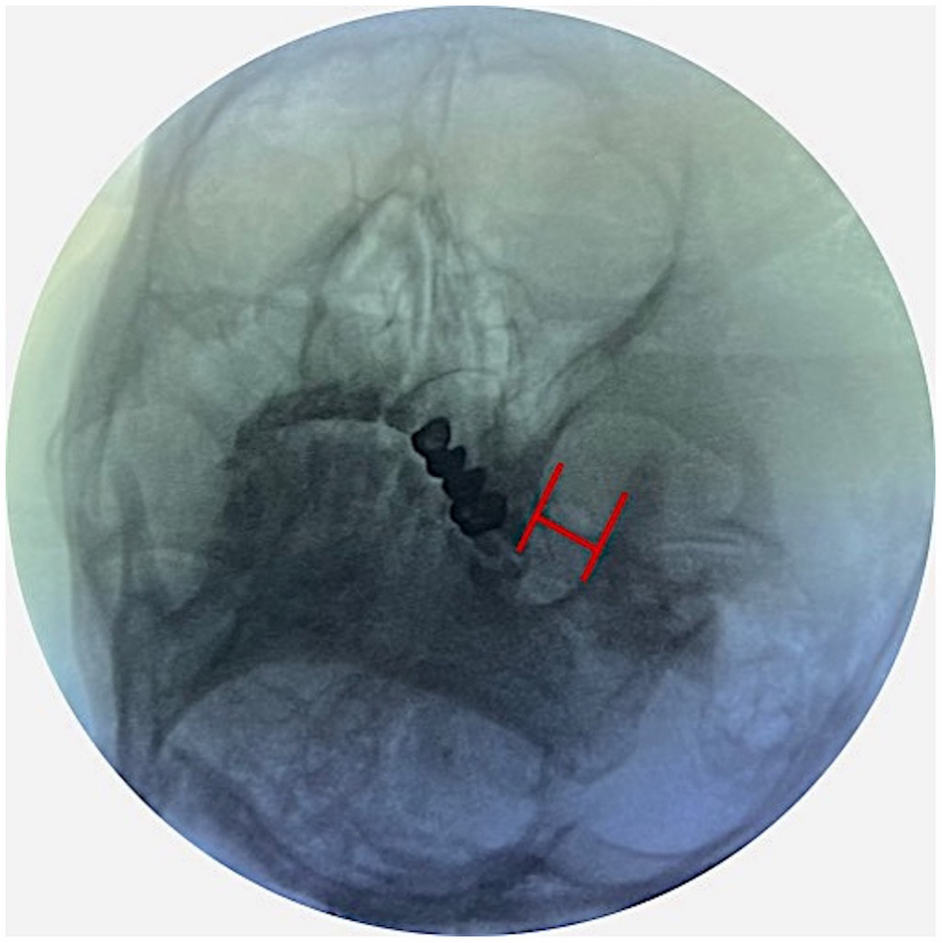
The fluoroscopic landmark of the H-figure to identify foramen ovale.

Patients were placed in a supine position with a 10 cm cushion placed under the shoulder, allowing for a slight neck extension. The initial fluoroscope intensifier (General Electric OEC 9900 Elite C-Arm; Bluestone Diagnostics) was positioned with 15° of lateral rotation from the midline toward the ipsilateral side and 15° of caudal tilt. The H-figure method was used to identify foramen ovale (8) (Figure 1).

**Figure 2:**
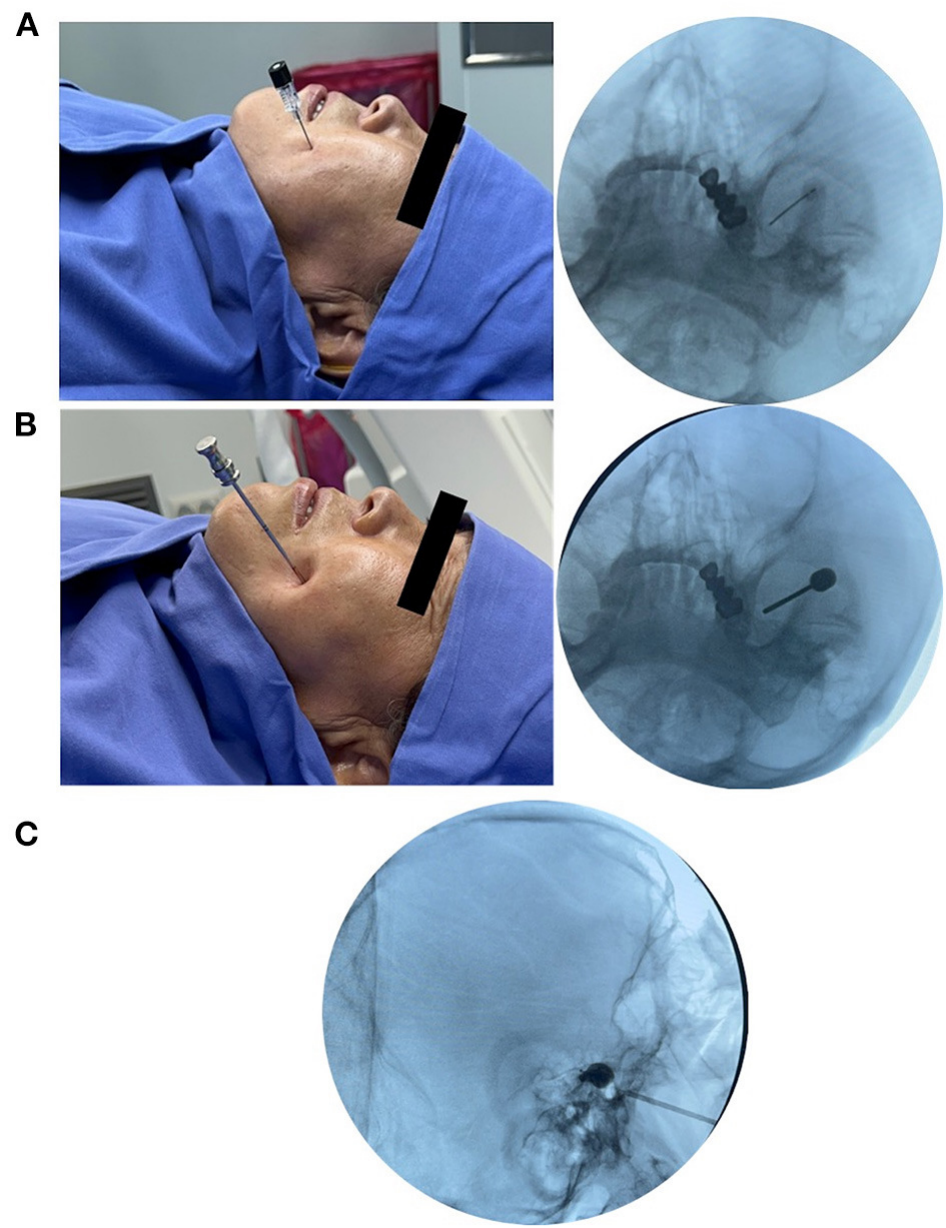
Percutaneous balloon compression procedure. **(A)** Local anesthesia of trigeminal ganglion with a 22-G needle. **(B)** Cannulation with a 14-G cannula. **(C)** Balloon compression with a No. 4 Fogarty catheter.

After localization of foramen ovale, a 10 cm 22-G needle was inserted through the sterilized skin in the “Haertel” approach (9) after local tissue infiltration with 5–10 mL 0.5% lidocaine and intravenous injection of 5 μg sufentanil and 0.5 mg atropine. Under fluoroscopic guidance, the needle tip was advanced through the medial half of the foramen ovale (Figure 2A). Thereafter, 1 mL of 0.25% lidocaine was injected into Meckel's cave to complete the local block of the trigeminal ganglion. The successful completion of local anesthesia of the gasserian ganglion is indicated by the subjective numbness in the distribution area of the affected trigeminal nerve and mild reduction of superficial sensation. When the needle was withdrawn, 2–3 mL of lidocaine was injected around the edge of the foramen ovale to reduce the pain associated with subsequent cannulation. After infusing local anesthesia, a 14-G cannula was inserted into the foramen ovale from the same path as the anesthesia needle used before (Figure 2B). After cannulation, a balloon catheter (Shenzhen Qingyuan Medical Equipment Co., Ltd, China) was inserted into the trigeminal cistern and inflated with a contrast agent to compress the trigeminal nerve (Figure 2C). The final balloon volume ranged between 0.35 and 0.8 mL and was adjusted based on the pressure and shape of the balloon. Local anesthesia was administered during the procedure to ensure patient comfort and safety. To assess the degree of nerve compression in real time, the surgeon interacted with the patient. Additionally, a sterile cotton ball was used to gently touch the skin of the patient's face to evaluate the degree of superficial sensory impairment. When the patient reported a complete loss of sensation to light touch on the affected side but could still perceive slightly stronger stimuli, the balloon compression was stopped. This ensured that an appropriate level of nerve compression had been achieved.

Mean arterial pressure (MAP), heart rate (HR), and VAS were measured by nursing staff in the operation room six times perioperatively, namely, during skin infiltration anesthesia (T0), local anesthesia needle puncture to foramen ovale (T1), at the time of injection of local anesthetics into trigeminal ganglion (T2), cannula insertion into foramen ovale (T3), balloon compression (T4), and surgery completion (T5). Each patient will undergo pain and numbness assessment, as well as a physical examination to evaluate the degree of hypoesthesia and other complications at discharge (2 days after surgery).

Long-term follow-up was carried out at 1, 3, 6, and 12 months by telephone interviews or outpatient clinic visits. Pain intensity was assessed using a VAS score from 0 to 10 (0 rated as no severity and 10 as worst severity). Pain relief was considered when the VAS score was < 4. For facial numbness, the degree of facial numbness was assessed by the Barrow Neurological Institute (BNI) facial hypesthesia scale (10): Class I, no facial numbness; Class II, mild facial numbness and not bothersome; Class III, facial numbness and somewhat bothersome; and Class IV, facial numbness and very bothersome. The Patient Satisfaction Index (PSI) score is divided into four levels, ranging from 1 to 4. 1 = surgery/therapy met my expectation; 2 = I did not improve as much as I had hoped, but I would undergo the same operation/ therapy for the same results; 3 = surgery/therapy helped, but I would not undergo the same operation/therapy for the same outcome; and 4 = I am the same or worse compared to before surgery/therapy.

### Statistical analysis

All data were processed with statistical analyses performed using SPSS software, version 23.0 (IBM SPSS, New York, NY, USA), and statistical significance was declared at the level of *P*-value of < 0.05 (two-tailed). Normally distributed continuous data on patient demographics and TN characteristics were reported as the mean and standard deviation, and non-normally distributed continuous data were reported as a median (25th quartile to 75th quartile). One-way repeated-measures analysis of variance was used to compare the VAS score HR, and MAP at different time points. The Kruskal–Wallis test was used to evaluate the rating of facial numbness in 12 months of follow-up.

## Results

A total of 87 patients underwent percutaneous balloon compression of the trigeminal ganglion with local anesthesia. The baseline characteristics of the patients are summarized in Table 1.

**Table 1:** Demographic characteristics.

**Age (mean ± SD) (y)**	**64.76 ± 11.65**
**Sex**, ***n*** **(%)**	
Male	25 (28.7%)
Female	62 (71.3%)
**Affected side**, ***n*** **(%)**	
Left	31 (35.6%)
Right	56 (64.4%)
**Affected nerve branch**, ***n*** **(%)**	
I	2 (2.3%)
II	18 (20.7%)
III	21 (24.1%)
I + II	7 (8.0%)
II + III	35 (40.2%)
I + II + III	4 (4.6%)
VAS score (mean ± SD)	7.65 ± 1.31
Disease duration (mean ± SD)(y)	6.62 ± 4.56

**Figure 3:**
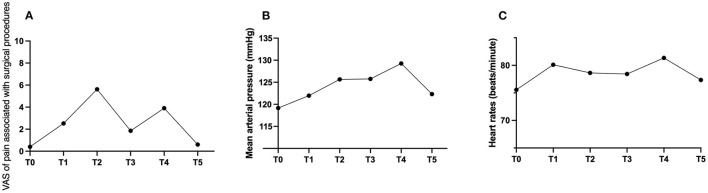
**(A–C)** Graphs showing the perioperative changes in VAS, mean arterial pressure (MAP), and heart rate.

The intraoperative VAS (pain associated with surgical procedures) demonstrated a statistically significant increase from T1 to T2 and a decrease from T2 to T3, T4, and T5 (*p* < 0.001) (Figure 3A). The most intense pain occurs at the time of injection of local anesthetics into the trigeminal ganglion (5.62 ± 3.58). The perioperative MAP and HR are displayed in Figures 3B, C. The results of the repeated measures ANOVA showed significant differences in MAP and HR among the six time points during the surgery (*F* = 4.51, *P* < 0.001). Pairwise comparisons revealed that the MAP during the puncture and balloon compression (T1-T4) was significantly higher than that during the infiltration anesthesia (T0). The MAP during balloon compression (T4) was significantly higher than that at other time points (*P* < 0.001). The analysis of HR showed similar results, with HR during balloon compression (T4) being significantly higher than at other time points (*P* < 0.05). However, throughout the entire surgery, the fluctuations of MAP and HR were within 20% of the baseline value (T0), and there was no circulation inhibition caused by the trigeminal reflex.

VAS scores of TN were significantly lower at discharge and 1-, 3-, 6-, and 12-month follow-up compared with pre-surgery (*P* < 0.001) (Table 2).

**Table 2:** Different time of VAS scores.

	**Mean ± SD**	***t* (paired *t*-test with baseline)**	***p*-value**
Baseline	7.65 ± 1.31		
Discharge	1.52 ± 1.11	33.30	< 0.001
1 month	0.97 ± 1.62	29.91	< 0.001
3 months	1.15 ± 1.95	25.81	< 0.001
6 months	1.14 ± 2.06	24.87	< 0.001
12 months	1.66 ± 2.41	20.37	< 0.001

**Figure 4:**
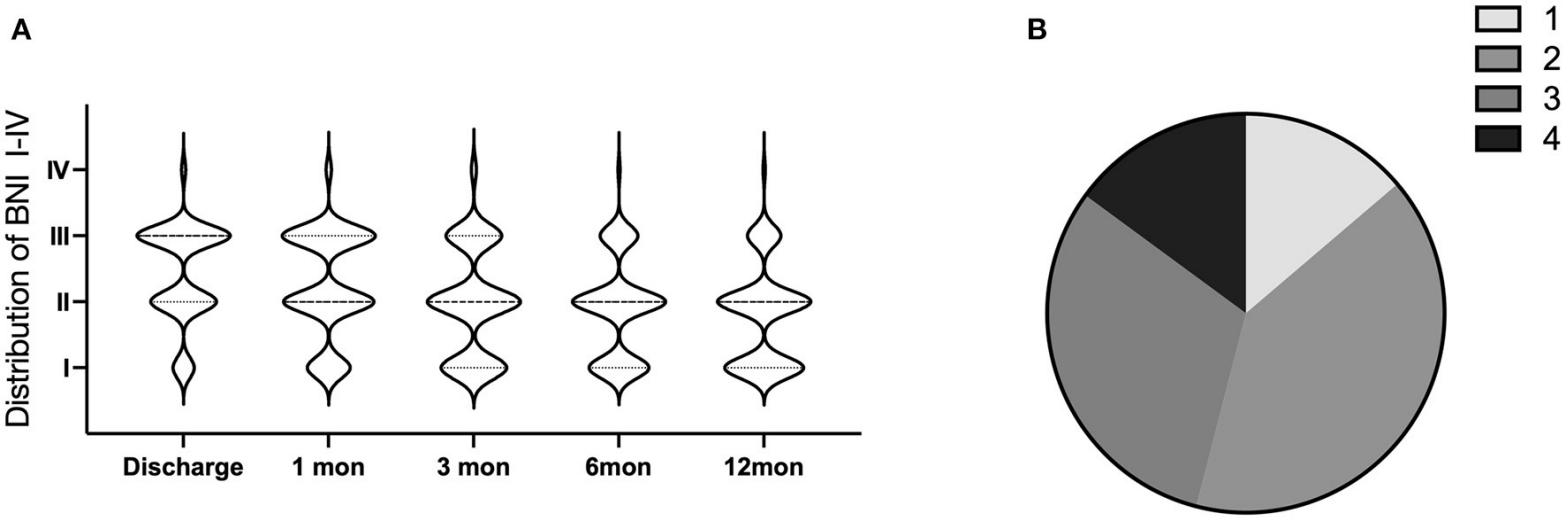
The outcomes after balloon compression. **(A)** The distribution of facial numbness BNI rating in 12 months follow-up. **(B)** The percentage of Patient Satisfaction Index (PSI) at postoperative 12 months (1 = surgery/therapy met my expectation; 2 = I did not improve as much as I had hoped, but I would undergo the same operation/therapy for the same results; 3 = surgery/therapy helped, but I would not undergo the same operation/therapy for the same outcome; and 4 = I am the same or worse compared to before surgery/therapy).

The rating of postoperative facial numbness (BNI I-IV) is shown in Figure 4A. After 1 year of surgery, the proportion of moderate to severe numbness was only 17.24%. While at postoperative 12 months, according to the PSI score, the number of patients who reported scores 1, 2, 3, and 4 was 12 (13.3%), 35 (40.8%), 27 (31.6%), and 13 (14.3%), respectively (Figure 4B).

A total of seven patients experienced ecchymoma at the needle insertion site, four patients complained of weakness of the masticatory muscles, and two patients developed herpes simplex 1 week after surgery. All patients fully recovered by postoperative week two.

## Discussion

As compared to MVD surgery, percutaneous minimally invasive procedures such as percutaneous trigeminal nerve ganglion radiofrequency thermocoagulation and percutaneous balloon compression can result in sensory nerve loss. However, these procedures provide an effective and relatively safe option for the management of trigeminal neuralgia, making it a preferred choice for both patients and healthcare professionals (11, 12).

A typical “pear” shape of balloon is a crucial imaging indication to ensure the efficacy of PBC. The accurate placement of the balloon in Meckel's cave can be achieved using imaging guidance under either general anesthesia or local anesthesia. General anesthesia can undoubtedly improve the patient's intraoperative experience and provide better operating conditions for the surgeon (13). Therefore, in the past, the majority of PBC surgeries were performed under general anesthesia with endotracheal intubation or laryngeal mask placement (14).

As patients with trigeminal neuralgia are often elderly and have underlying diseases, general anesthesia can be contraindicated. Additionally, trigeminal ganglion compression under general anesthesia can trigger a life-threatening trigeminal-cardiac reflex, resulting in a significant decrease in blood pressure, heart rate, and even cardiac arrest. Stimulation of the trigeminal ganglion sends afferent signals to the sensory nucleus of the trigeminal nerve near the floor of the fourth ventricle. Small internuncial nerve fibers in the reticular formation connect afferent to efferent premotor neurons primarily located in the nucleus ambiguous and the dorsal motor nucleus of the vagus. The reflex pathway then activates cardioinhibitory parasympathetic vagal neurons, thereby completing the reflex arc (15).

Recent studies have demonstrated that local anesthetic infiltration or nerve block of the afferent pathway may prevent TCR (4, 16). Tibano et al. conducted a study in which patients were divided into a deep sedation plus trigeminal ganglion block anesthesia group (injection of 2% lidocaine 0.8 ml) and a deep sedation plus trigeminal ganglion injection of the same volume of the saline control group for balloon compression (4). The results showed that the changes in blood pressure and heart rate were more stable in the trigeminal ganglion block group than in the control group. In our study, we found that although the VAS score for surgery-related pain was significantly higher during the puncture process than before surgery, the heart rate and blood pressure did not fluctuate significantly compared with the preoperative values. Additionally, during the operation, no patient requested to switch to general anesthesia due to severe pain.

In this study, we investigated facial numbness as a primary evaluation indicator for balloon compression, as it can significantly impact a patient's quality of life, especially in the case of neurectomy-related facial numbness such as in trigeminal neuralgia. Furthermore, in addition to balloon shape, balloon pressure and compression time are important factors affecting postoperative efficacy, which cannot be customized under general anesthesia (17). As a result, many patients are unable to achieve the desired level of nerve damage, which is crucial for long-term pain relief without excessive numbness.

Operating with the patient awake enables the surgeon to assess the degree of nerve damage in real time and helps to avoid excessive facial numbness after surgery and complications such as corneal ulcers. Our study demonstrated that none of the patients developed severe facial numbness, irreversible keratitis, decreased vision, or weakness of the masticatory muscles. Furthermore, the majority of patients reported satisfaction with the treatment.

There are some limitations that should be acknowledged. First, we did not conduct neuroelectrophysiological tests, such as evoked potentials, after the procedure. As a result, our evaluation of postoperative numbness relied mostly on subjective patient descriptions and lacked objective data. Second, randomized clinical controlled trials would be more suitable than our case series study for evaluating the outcomes of balloon compression under conscious sedation of local anesthesia for trigeminal neuralgia as they can provide more accurate results.

## Conclusion

Our study demonstrates that percutaneous balloon compression of trigeminal ganglion under conscious sedation local anesthesia is an effective minimally invasive procedure for the treatment of primary trigeminal neuralgia.

## Data availability statement

The raw data supporting the conclusions of this article will be made available by the authors, without undue reservation.

## Ethics statement

The studies involving human participants were reviewed and approved by Capital Medical University Xuanwu Hospital Ethics Committee. The patients/participants provided their written informed consent to participate in this study. Written informed consent was obtained from the individual(s) for the publication of any potentially identifiable images or data included in this article.

## Author contributions

DZ helped design the study, conduct study, analyze data, and prepare the article. YG and LH helped collect data. LY helped revise the article. All authors have read and approved the manuscript.
